# Born to Speak and Sing: Musical Predictors of Language Development in Pre-schoolers

**DOI:** 10.3389/fpsyg.2019.00948

**Published:** 2019-05-24

**Authors:** Nina Politimou, Simone Dalla Bella, Nicolas Farrugia, Fabia Franco

**Affiliations:** ^1^Department of Psychology, Middlesex University, London, United Kingdom; ^2^International Laboratory for Brain, Music and Sound Research (BRAMS), Montreal, QC, Canada; ^3^Department of Psychology, University of Montreal, Montreal, QC, Canada; ^4^Centre for Research on Brain, Language and Music (CRBLM), Montreal, QC, Canada; ^5^Department of Cognitive Psychology, University of Economics and Human Sciences in Warsaw, Warsaw, Poland; ^6^Lab-STICC, Department of Electronics, IMT Atlantique, Brest, France

**Keywords:** musical skills, language development, pre-school children, informal musical experience, home environment

## Abstract

The relationship between musical and linguistic skills has received particular attention in infants and school-aged children. However, very little is known about pre-schoolers. This leaves a gap in our understanding of the concurrent development of these skills during development. Moreover, attention has been focused on the effects of formal musical training, while neglecting the influence of informal musical activities at home. To address these gaps, in Study 1, 3- and 4-year-old children (*n* = 40) performed novel musical tasks (perception and production) adapted for young children in order to examine the link between musical skills and the development of key language capacities, namely grammar and phonological awareness. In Study 2, we investigated the influence of informal musical experience at home on musical and linguistic skills of young pre-schoolers, using the same evaluation tools. We found systematic associations between distinct musical and linguistic skills. Rhythm perception and production were the best predictors of phonological awareness, while melody perception was the best predictor of grammar acquisition, a novel association not previously observed in developmental research. These associations could not be explained by variability in general cognitive functioning, such as verbal memory and non-verbal abilities. Thus, selective music-related auditory and motor skills are likely to underpin different aspects of language development and can be dissociated in pre-schoolers. We also found that informal musical experience at home contributes to the development of grammar. An effect of musical skills on both phonological awareness and language grammar is mediated by home musical experience. These findings pave the way for the development of dedicated musical activities for pre-schoolers to support specific areas of language development.

## Introduction

Music, like language, is a highly complex system. In language smaller units such as phonemes and morphemes are combined to form higher-order structures, namely words and sentences. Similarly, in music, separate units (pitches and durations) are combined to form higher-order sequences such as musical phrases and compositions. Musical and linguistic sequences both contain melodic and rhythmic patterns: melody and rhythm in music, and prosody in language (Patel, 2010).

Commonalities between music and speech are evident when looking at infant perception and production of sounds and mother–infant communication. The remarkable sensitivity of infants to melodic features like contour and pitch changes (e.g., Trehub et al., 1985) may arise from the early auditory, *in utero* experience of maternal speech (Kisilevsky et al., 2004; Mampe et al., 2009). Notably, important musical elements of infant vocalizations during the first year of life, namely melodic and temporal patterns (D’Odorico et al., 1985; D’Odorico and Franco, 1991), and manner of phonation (Franco, 1984) are produced in specific communicative contexts (see also Papaeliou and Trevarthen, 2006). The distinctive way adults speak when addressing infants, also known as *Infant Directed* (*ID*) speech or *“motherese”* (Fernald, 1985; Fernald and Kuhl, 1987), is characterized by higher pitch and exaggerated rhythmic and melodic patterns (Fernald, 1985; Trehub et al., 1993). This richly intonated type of speech that efficiently conveys the prosodic features of one’s native language may shape infant vocal production in the first year of life. Indeed it has been argued that not only infants’ early vocal “musical” behaviors are directly linked to the prosodic characteristics of their native language (Ruzza et al., 2003; Welch, 2006) but also that newborn cry vocalizations imitate their surrounding native speech prosody (Mampe et al., 2009). Interestingly, the capacity to process speech prosody and to perceive intonational contours in melody is an area where music and language overlap in the brain (Patel, 2010).

The discovery of early musical abilities in infants has also provided fruitful ground for the study of music and language from a phylogenetic perspective (Cross, 2003; Tomlinson, 2013). Similar to language, infants appear to be immediately sensitive to music (e.g., Trehub et al., 1985). This inclination is later shaped by human interaction in the context of culture-specific forms, i.e., different languages and different musical systems. Shared features between language and music have led to the hypothesis that a song-like communication system may be the phylogenetic precursor of modern language (Darwin, 1871; Livingstone, 1973; Brown, 2001; Merker, 2002; Mithen, 2005). Although there is still much debate about the adaptive function of musical skills in evolutionary terms (Fitch, 2006, for a discussion), some interesting observations of human infants also point to deep connections between musicality and early communication development. For example, child-directed singing has been shown to play an important role in regulating infant arousal and in establishing the mother–infant emotional bond (Trehub and Trainor, 1998; Trevarthen, 1999; Shenfield et al., 2003). Moreover, infants display superior attention to sung than spoken infant-directed communication (Nakata and Trehub, 2004; Tsang et al., 2017). Similarly, musical characteristics of mother–infant vocal interactions are critical in promoting the development of socio-emotional regulation (Trevarthen, 1999; Dissanayake, 2000; Van Puyvelde et al., 2014). Finally, the acquisition of musical and linguistic features such as rhythm entrainment and vocal learning may rely on common learning mechanisms with adaptive value for social development, such as imitation (Carpenter, 2006; Fitch, 2013; Laland et al., 2016). In sum, interesting comparisons can be drawn between the phylogeny of language and music when focusing on their role for early communication development.

Accordingly, linguistic and musical skills are expected to emerge in parallel and to be linked across early development. Evidence from previous studies points in this direction. Randomized controlled trials (RCTs) with school-aged children have revealed language-related advantages for children participating in music groups (Moreno et al., 2009; François et al., 2013; Kraus et al., 2014). For example, after 2 years of participation in music versus painting classes, a music group outperformed controls on both electrophysiological and behavioral measures of speech segmentation (i.e., ability to extract pseudo-words from a continuous stream of nonsense syllables) (François et al., 2013). In another study, 8-year-old children receiving conventional musical training for 24 weeks showed enhanced performance in both reading measures and pitch discrimination abilities in speech (Moreno et al., 2009). Notably, these differences cannot be accounted for by pre-existing traits in musicians, like general IQ (Schellenberg, 2004), as such variables were controlled in these experiments.

Pertinent research with pre-schoolers has been scarce. Two RCTs with 4- to 6-year-old children comparing the effects of musical training to other types of training reported enhancements in phonological awareness skills (Degé and Schwarzer, 2011, with 5- and 6-year-olds), and vocabulary (Barac et al., 2011, with 4- and 6-year-olds). These findings suggest that features of music instruction may strengthen aspects of linguistic development in young children. In a correlational study Anvari et al. (2002) showed that both rhythmic and melodic aspects of musical ability were associated with phonological awareness and early reading ability (early identification of letters and reading small phrases) in 4-year-old children. Other researchers have shown that pitch discrimination contributes to phonological awareness in 4.5- to 6-year-old children (Lamb and Gregory, 1993; Forgeard et al., 2008), while a link between phonological awareness and rhythmic abilities was observed in 5-year-old children (Anvari et al., 2002; Verney, 2013). Only one study so far has investigated a link between rhythmic abilities and phonological awareness in children younger than 4 years (Woodruff Carr et al., 2014). In this study, synchronization to an external beat was linked to speech encoding and phonological awareness in 3- and 4-year-old children. Taken together the results from correlational and longitudinal studies indicate that specific associations between musical and linguistic abilities may be present in pre-school children.

The potential involvement of pitch and melody perception in the language development of children younger than 4 years old remains unclear. Melody and pitch are critical elements of speech prosody, influencing language learning mechanisms such as statistical learning (Saffran et al., 1996; Thiessen et al., 2005), and conveying crucial information that aids speech segmentation and linguistic pattern extraction (Brooks and Kempe, 2012; Xie, 2012). Furthermore, aspects of linguistic development other than phonology, like the development of morphological rules and grammar have been neglected in studies with younger age groups. Only one study of typical children shows that rhythm perception skills and language structure and morphology are related in 6-year-old children (Gordon et al., 2015). Yet, infants and children are likely to rely on both rhythmic and melodic prosodic cues to extract grammatical structures. For example, changes in pitch tend to correspond to boundaries between different syntactic clauses and phrases (Brooks and Kempe, 2012) aiding the extraction of grammatical information from continuous speech.

In sum, associations between linguistic and musical skills are observed quite systematically in children older than 4 years. Findings show that formal musical training generates advantages for auditory processing and language skills. Commonalities in the perception of musical and linguistic sounds have also been researched in infancy. However, a comprehensive account of the relationship between music and language across the full developmental spectrum is lacking. There is a significant knowledge gap for children between 1 and 4 years of age. Filling this gap is paramount to examine the effects of specific music-oriented training on early years education.

The first goal of this study is therefore to shed light on the developmental trajectory of the relationship between musical skills and different aspects of language development in 3- and 4-year-old children. To this aim, in Study 1, children were submitted to tests of both phonological awareness and grammar, two areas of language development that have been linked to the successful acquisition of literacy skills and academic attainment (Deacon and Kirby, 2004; Melby-Lervåg et al., 2012; Cunningham and Carroll, 2015). Notably, these abilities have been treated as prerequisites for the development of fine-grained aspects of language production and understanding, such as pragmatics (Hoff and Marilyn, 2009). To pinpoint the musical skills relating to these key areas of language development we used a range of musical measures, assessing pitch, melody, rhythm, and tempo perception, singing and synchronization to the beat. In line with previous studies in older children we hypothesized that musical and linguistic skills would be linked in young pre-schoolers.

Testing musical skills in young pre-schoolers (3- and 4-year-olds), so far mainly neglected, poses methodological challenges. The typical testing environment combined with the auditory nature of the stimuli requires a level of attention that is difficult for such young children to maintain. Musical testing in this age group has for a long time relied on *Audie’s* test (Gordon, 1989), which measures musical perception abilities but only includes two subtests, melody and rhythm discrimination, excluding other aspects of music perception important for musical expression and performance such as tempo (Law and Zentner, 2012) or basic auditory perceptual abilities such as pitch discrimination. Furthermore, it does not include any music production tasks, which can be rich sources of information for measuring a child’s musical ability. To achieve the goals of Study 1 we developed a set of novel musical tests of melody and rhythm perception/production adapted specifically for this age group.

Another largely unexplored area of research is the influence of informal musical activities at home on musical and linguistic development. Notably, most pre-school children experience music through informal interaction at home rather than receiving formal music lessons. Only two studies so far (Putkinen et al., 2013; Williams et al., 2015) have directly assessed the effect of informal home musical experience on language development. They reported enhanced language and music-related auditory processing (Putkinen et al., 2013) and improved vocabulary (Williams et al., 2015) in young pre-schoolers as a function of informal musical experience at home. Although these findings are important, they remain fragmentary and a more systematic examination of the effect of this type of experience on key musical and linguistic skills is lacking. Such findings can inform early childcare practice both in the family and in educational contexts. They also have theoretical implications for the role of environmental factors in the development of children’s abilities.

The objective of Study 2 was therefore to examine whether informal musical interactions and experience within the family might have an impact on children’s musical and linguistic skills as assessed in Study 1. Based on previous findings we hypothesized that this aspect of environmental input would have a specific influence on the development of key linguistic areas (i.e., phonological awareness and grammar) and musical abilities, as assessed in Study 1.

## Study 1: Links Between Musical and Linguistic Skills

### Materials and Methods

#### Participants

Forty pre-school children (21 boys) between the ages of 3 years and 5 months and 4 years and 9 months (*M_age_* = 4 years, *SD* = 4.7 months) were recruited from nursery classes. Twenty-eight children were monolingual English speakers and 12 children were bilingual but had English as their first language (as reported by the parents). A language comprehension test (British Picture Vocabulary Scale; Dunn et al., 1997) was administered to all participants to ensure that they possessed an adequate level of English comprehension for their age. None of the participants had hearing difficulties or had been diagnosed with developmental delays. All of the children experienced comparable musical activities in their nursery as reported by their teachers. Five children also received music-related training (dancing or singing) outside the home.

#### Tests of Musical Abilities

An original battery of age-appropriate musical tests was designed for this study. The battery included both music perception and music production tasks. Music perception tasks assessed pitch, melody, rhythm, and tempo perception. Music production tasks tested singing and tapping to a beat. Extensive piloting served to evaluate the feasibility of these tasks for young children.

##### Music perception

**FIGURE 1:**
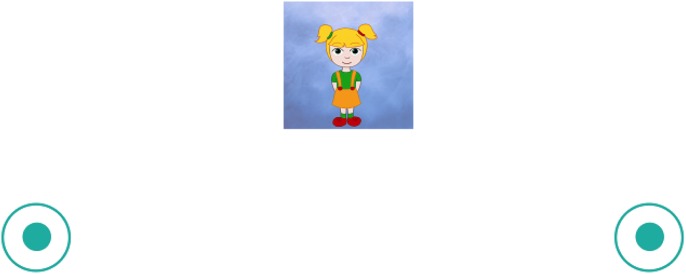
Visual configuration appearing in the music perception tasks (shapes and colors differ among trials).

Music perception tasks (pitch, melody, rhythm, and tempo perception) used a 2 + 1 oddity paradigm (e.g., Jensen and Neff, 1993). This method is well suited for non-verbal assessment of auditory discrimination in young children (White et al., 1990; Jensen and Neff, 1993), as confirmed in our pilot study. In each trial the child listened to a musical stimulus (i.e., melody or pitch) corresponding to the drawing of a little girl named Maggie that appeared at the top of the computer screen (see Figure 1). Two identical shapes would then appear successively on the lower left and right sides of the screen corresponding to two musical stimuli. One was the same as Maggie’s stimulus, the other was different. The child was asked to point to the shape that sounded the same as Maggie’s stimulus. Stimulus position on the screen (left or right) and order of appearance (whether the same item would be heard first or second) was counterbalanced. All stimuli were presented at 75 db (Jensen and Neff, 1993). Stimuli in each trial were separated by 1-s silent intervals. Inter-trial intervals varied in order to ensure that the child was attentive before each trial. Order of trials in all tasks was randomized across participants. Positive and negative feedback was provided to increase motivation. All tasks were designed and run using E-prime software.

To ensure that the children fully understood the music perception task format, in the first session they received four practice trials including easily identifiable sound stimuli (e.g., a dog barking). The child had to complete three out of four trials correctly. Participants were excluded from the study if they did not meet this criterion. Only one child failed to reach the criterion. Three practice trials also preceded each music perception task.

###### Single pitch perception

The stimuli (*n* = 10) in the pitch perception task were pure tones (duration = 400 ms; with 25-ms linear onset and offset ramps), generated with *Audacity* software. The pitch of the standard stimulus (1,000 Hz) was chosen as infants’ discrimination ability is superior for high rather than low frequencies (Olsho, 1984), and it has been used as a reference frequency in a number of experiments both with children (e.g., Thompson et al., 1999; Agnew et al., 2004; Bobin-Bègue and Provasi, 2005) and with adults (e.g., Tillmann et al., 2003; Paraskevopoulos et al., 2012; Weiss et al., 2014). Comparison stimuli differed in frequency (lower or higher) and hovered around 1,000 Hz. Fifty percent of the comparison stimuli had a lower pitch, while the remaining 50% had a higher pitch compared to the standard stimulus. The first comparison stimulus represented the easiest trial and differed from the standard by 120 Hz while the difference between comparison and standard stimuli in the remaining nine trials ranged from 12 to 60 Hz.

###### Melody perception

Six 3-tone melodies and six 5-tone melodies (1–3 s) were composed for the melody perception task. Each melody was originally composed in C major but was then transposed into a different musical key (all major scales were used). Melodies were played at a tempo of 140 BPM, which is close to the spontaneous motor tempo of children in this age group (Provasi and Bobin-Bègue, 2003).

**FIGURE 2:**
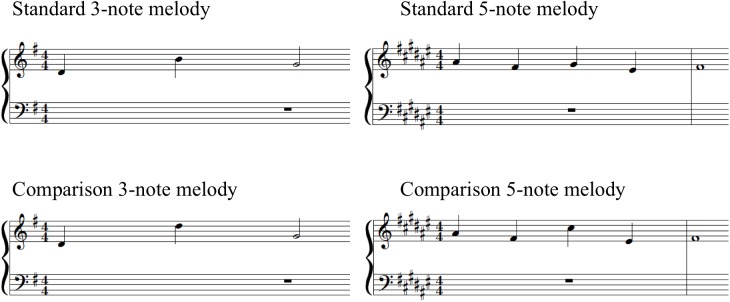
Musical notation for standard and comparison versions of one example 3-note melody and one example 5-note melody.

Differences to be detected in the comparison stimuli consisted of one-tone changes. Difficulty was manipulated across two levels: (a) the length of the melodies (stimuli included six 3- and six 5-note melodies) and (b) changes in comparison stimuli were either contour-violating or contour-preserving. This was based on previous work showing that 4- to 6-year-old children can more readily identify contour-violating compared to contour-preserving transformations in short melodies (Morrongiello et al., 1985). All changes in comparison melodies were limited to one tone in the middle of the melody (i.e., second tone in 3-note melodies and second, third, or fourth tone in the 5-note melodies). Changes did not violate the key of the standard melody. Pitch interval changes ranged from 3 to 12 semitones and included both upward and downward changes. The average pitch interval changes were equivalent across 3- and 5-note melodies with a mean of 6.5 (range: 3–12) and 6.2 (range: 3–12) semitones, respectively. Average pitch interval changes were, however, different between contour-violating (mean of 9.3 and range of 8–12 semitones) and contour-preserving stimuli (mean of 3.8 and range of 3–5 semitones). Figure 2 shows the musical notation for standard and comparison versions of one example 3-note melody and one example 5-note melody.

###### Rhythm perception

The same melodies from the melody discrimination task were used in this task. Differences to be detected in the comparison stimuli consisted of changes in the duration of adjacent tones. This manipulation altered the rhythmic grouping of the comparison melody while preserving the number of notes and the overall duration and meter of the standard melody (see also Peretz et al., 2013). Difficulty of the stimuli was manipulated (a) by varying the length of the melodies (as in the melody discrimination task), (b) by changing duration of either two or three tones in a sequence, and (c) by introducing changes in duration occurring either on the downbeat (easy trials, 50% of stimuli) or on the upbeat (more difficult trials, 50% of stimuli) of the melody’s meter.

###### Tempo perception

Ten 4-note melodies were used in the tempo perception task. Four, rather than 3-note melodies (as in part of the rhythm and melody discrimination tasks) were used to ensure that the children adequately recognized the tempo of each melody. All melodies were composed in the key of C major and were then transposed into different musical keys (10 major scales). The tempo of all standard stimuli was 100 BPM. The rate of the standard stimuli was chosen based on previous experiments showing that this is the optimal sensitivity zone for tempo discrimination in both infants (Baruch and Drake, 1997) and adults (Baruch et al., 2004). Furthermore, the only study that has so far examined tempo discrimination thresholds in children of 3- and 4-years of age has used 100 BPM as a the standard tempo (Bobin-Bègue and Provasi, 2005). Based on the above study and on pilot observations, the differences in tempo in the final set of stimuli ranged from 25 to 70 BPM (around the standard tempo).

##### Music production

###### Song production

For the evaluation of song production children were asked to sing a popular children’s song (“Twinkle Twinkle Little Star”) along with the voice of the experimenter that was pre-recorded and played through a portable speaker at a tempo of 100 BPM [the instrumental part of the recording was taken from Verney (2013)]. Using *Audacity* software and a Zoom H4n audio interface the child’s voice was recorded on a MacBook Pro laptop.

Two musically trained independent raters evaluated the performance of the children. Evaluation was based on two rating scales developed by Rutkowski (1997; Singing Voice Development Measure) and Welch (1998, 2006). These rating scales were based on two longitudinal studies of children’s singing development starting from pre-school years, which led to models featuring different phases of early vocal development. Taken together, these models suggest that children’s performance progresses from singing that is centered on the words of the song (chant-like singing) to singing within a limited pitch range that follows the contour of the target melody.

Rating of the songs involved identifying the specific pitch range produced by the child and comparing it to the recorded song. Pitch range was identified using *Audacity*, which provides an accurate estimation of the range in fundamental frequency. The Rutkowski’s (1997) scale was slightly adjusted to include an extra category (i.e., “Melodic shape exists and follows the contour of the original. There is some variability in pitch not necessarily accurate but following the correct contour”) that proved to be useful in the evaluation of the sample. A mean score of the two evaluations was calculated (range 0–10) and entered in all subsequent analyses. Inter-observer agreement was very high as shown by the high correlation between observers’ ratings (*r* = 0.91, *p* < 0.001).

###### Synchronization to the beat

The ability to move to a beat was assessed with a synchronized tapping task. In this task, children were asked to tap along to the sounds of a metronome, played at two tempi (120 BPM and 100 BPM) in two separate trials. The beat (metronome clicks) was played by an animated avatar on a computer screen. The specific tempi were chosen based on previous work suggesting that pre-school children synchronize with stimuli presented at fast tempi ranging from 100 to 150 BPM (Provasi and Bobin-Bègue, 2003). As each child has a unique preferred tempo, this is unlikely to be captured by one of the two tapping rates; calculating the mean of the two tapping rates seemed more appropriate to reflect the participants’ performance (see Woodruff Carr et al., 2014). Children’s tapping was recorded using a custom contact microphone that was inserted inside a toy drum. A sampling rate of 44,100 Hz was used to record the audio from the contact microphone.

The onsets of the taps (*n* = 20) were identified using the audio signal from the contact microphone, processed using a semi-automatic method. Each sample was squared and compared to a default threshold set to four times the standard deviation of the squared trial, and was marked as a candidate tap onset. A refractory window preventing the detection of a new tap onset in the 200 ms following each candidate tap onset was applied in order to only detect the first sample of each tap. This procedure was run on all trials and all subjects, and the results were visually inspected to ensure that each tap is detected only once, and that all taps were detected. Next, the two parameters (threshold for candidate taps and length refractory window) were manually adjusted relative to each participant’s tapping volume, and a final visual inspection was performed to validate all tap onsets. Deviations of each tap time from the corresponding metronome times (i.e., asynchronies) were calculated; the standard error of these asynchronies (SEA) indicated the variability of synchronization performance. Smaller SEA values indicate better synchronization performance (Table 1 shows a summary of music perception and production tasks).

**Table 1:** Summary of music perception and production tasks.

Musical tasks	Equipment	Stimuli	Participant task	No. of trials	Scoring
Pitch Perception	Computer	Sinusoids	Identify which of two pitches is the same as the standard	10	% correct
Melody Perception	Computer	Melodies	Identify which of two melodies is the same as the standard	12	% correct
Tempo Perception	Computer	Melodies	Identify which of two melodies is the same as the standard	10	% correct
Rhythm Perception	Computer	Melodies	Identify which of two melodies is the same as the standard	12	% correct
Song Production	Computer, microphone	Song recording	Sing along to recording of *Twinkle Twinkle Little Star*	1	Scored by two independent raters
Synchronization	Computer, drum, contact mic	Metronome clicks	Tap along to metronome clicks	100, 120 bpm	Standard error of asynchronies


#### Language

The Language Structure Index (LSI) from the Clinical Evaluation of Language Fundamentals - Preschool-2 (CELF-Preschool-2; Wiig et al., 2004) was administered to assess language grammar. The LSI consists of three subtests: Sentence Structure, Word Structure, and Recalling Sentences. Cronbach’s α reliability coefficients for the LSI and all the relevant subtests range from 0.78 to 0.94 (age range: 3.5–4.5 years). Standardized scores for the three subtests and the LSI were computed and used in all analyses.

Two subtests from the CELF-Preschool-2 were used to assess Word/Syllable blending and Sentence/Syllable segmentation. Two tests of rhyme and alliteration awareness – the Phonological Oddity – Rhyme and the Phonological Oddity – Alliteration task (Maclean et al., 1987) – replaced the rest of the phonological awareness subtests of the CELF-Preschool-2 (Rhyme perception and Rhyme generation), because during pilot testing these were deemed to be inappropriate for the younger, as well as some of the older children.

In order to derive a reliable composite score for phonological awareness and in absence of standardized scores for 3-year-old children, principal component analysis (PCA) was conducted on the four phonological awareness subtests. The Kaiser-Meyer-Olkin measure verified the sampling adequacy for the analysis: KMO = 0.68 (acceptable according to Kaiser, 1974). Bartlett’s test of sphericity, χ^2^ (6) = 33.1, *p* < 0.001, indicated that correlations between variables were sufficiently large for PCA. One factor had an eigenvalue exceeding Kaiser’s criterion of 1. All four subtests (Phonological Oddity – Rhyme, Phonological Oddity – Alliteration and Word-Syllable Blending and Sentence/Syllable Segmentation) loaded adequately onto that factor, which explained 55.89% of the variance (loadings ≥ 0.39). Therefore, factor scores for phonological awareness were used in subsequent analyses in addition to the separate subtests.

#### Memory and General Ability

The Digit Span subtest from the British Ability Scales II (BAS, Elliott et al., 1996) was used to assess verbal memory. Reliability coefficients for the Digit Span were calculated using the Hoyt (1941) formula and were 93 and 83 for 3- and 4-year-olds, respectively. The Block Design from the Wechsler Preschool and Primary Scale of Intelligence IV (WPPSI-IV, Wechsler, 2013) was used as a proxy for non-verbal ability (split-half reliability coefficient for 3- and 4-year-olds = 0.84).

#### Procedure

All participants were tested during six or seven individual sessions spread across 6–7 days, each lasting approximately 20 min. Testing for each participant was completed within 2–3 weeks of their first session. Individual sessions took place in specified quiet rooms in the participating nurseries. The first session included four practice trials of the music perception task format in order to determine whether participants understood the instructions and were able to perform the task. One child failed to meet the criterion (see section “Music Perception”). Order of administration was held constant for all children, alternating music and language tasks. However, the number of tasks completed in each session varied depending on the child’s attention span and mood on the given day. Before the start of the sessions, the experimenter who later conducted the testing spent approximately 2 days in classrooms playing with the children, in order for them to become acquainted with her and to feel comfortable during testing.

#### Data Analysis

All data were analyzed using SPSS version 20.0 and R software environment (R Core Team, 2012). Bivariate correlations were performed between musical, linguistic and cognitive skills. To investigate potential predictive relationships between musical and linguistic abilities, data were entered into two separate linear regression models with phonological awareness and language grammar as the dependent variables. Measures of musical ability (Pitch, Rhythm, Tempo and Melody Perception, Song Production and Synchronization) were treated as predictors. Age and gender were also entered into the models in order to examine whether they contribute to the variance in linguistic abilities. The drop1() function in R was used to gradually eliminate variables with no significant contribution to the model. The reported models are those which are the most explanatory and parsimonious after progressively removing the different predictors (backward).

Residual plots were visually inspected for all models to test for the assumptions of normal distribution, linearity, and heteroscedasticity of the data. No obvious patterns were observed and residuals did not appear to deviate from a straight line in any of the models, therefore meeting the assumptions.

### Results

Means and standard deviations of participants’ group performance in all linguistic, baseline cognitive, and musical tasks are presented in Table 2.

**Table 2:** Participants’ performance in cognitive, linguistic, and musical tasks.

	Method of scoring	*N*^1^	Min	Max	Mean	Standard deviation
BPVS	*M* = 100, *SD* = 15	40	86	129	107.8	10.03
CELF-LSI	*M* = 100, *SD* = 15	37	21	95	66.81	19.73
Phon Aw	Range of scores = 1–100	35	16.2	81.4	51.56	21.38
Block Des	Range of scores = 1–19	39	8	14	10.87	1.73
Digit Span	*M* = 50, *SD* = 10	38	22	92	69.63	20.16

**Musical tasks**	**Range of scores**	***N*^1^**	**Min**	**Max**	**Mean**	**Standard deviation**

**Perception**						
Pitch	1–10	37	3	10	6.35	1.60
Melody	1–12	38	4	11	7.81	1.99
Tempo	1–10	37	3	10	7.18	1.74
Rhythm	1–12	38	5	12	8.60	2.19
**Production**						
Song	1–10	36	2.5	10	7.06	2.18
Synch	1.28–6.64	38	1.28	6.64	3.79	1.63


*BPVS, British Picture Vocabulary Scale; CELF-LSI, Clinical Evaluation of Language Fundamentals – Preschool – Language Structure Index; Phon Aw, phonological awareness composite score; Block Des, Block Design. ^*1*^Please note that *N* sizes differ, as 7 out of 40 children did not complete all the tasks included in the assessment.*

A series of non-parametric comparisons (Mann–Whitney and Kolmogorov–Smirnoff) were first performed between monolingual (*n* = 28) and bilingual children (*n* = 12) to identify any differences in linguistic or cognitive performance resulting from bilingualism. As there were no statistically significant differences between monolingual and bilingual children in any of the tasks, data from both monolingual and bilingual children were pooled for all subsequent analyses.

To examine the relations between musical and linguistic skills, correlations between the measures in the respective tasks were calculated (see Table 3). As can be seen, a number of significant relationships were observed between musical skills and subtests and composite scores of phonological awareness and grammar. Different musical abilities (rhythm and synchronization vs. melody) were associated with different linguistic tasks. Performance in temporal or rhythmic tasks was mostly linked with phonological awareness, whereas performance in the melody task was associated with grammar. Rhythm perception was also significantly associated with one of the language grammar subtests (*Recalling Sentences*) while melody perception was associated with one of the phonological awareness subtests (*Sentence/Syllable Segmentation*).

**Table 3:** Correlations between musical tasks and subtests of phonological awareness and language structure.

Language tasks		Pitch	Tempo	Melody	Rhythm	Song	Synch
Grammar	SS	0.16	0.17	0.27	0.10	0.19	0.13
	WS	0.09	0.17	0.35^*^	0.02	0.17	-0.17
	RS	0.25	0.12	0.37^*^	0.33^*^	0.12	-0.15
	Comp	0.09	0.23	0.43^***^	0.24	0.29^1^	-0.21
Phon Aw	W/S	0.15	0.40^*^	0.26	0.36^*^	-0.09	-0.20
	S/S	0.10	0.41^*^	0.41^*^	0.31^*^	0.04	-0.36^*^
	Rh	-0.27	0.26	0.17	0.45^**^	0.19	-0.32^*^
	Alit	0.09	0.35^*^	0.25	0.67^***^	0.21	-0.38^*^
	Comp	0.04	0.38^*^	0.29^1^	0.63^***^	0.20	-0.47^**^


*^∗^*p* < 0.05, ^∗∗^*p* < 0.01, ^∗∗∗^*p* < 0.001. ^*1*^Marginally significant (*p* < 0.1). SS, Sentence Structure, WS, Word Structure, RS, Recalling Sentences, W/S, Word/Syllable Blending, S/S, Sentence/Syllable Segmentation; Rh, Phonological Oddity Rhyme; Alit, Phonological Oddity Alliteration; Comp, Composite Score.*

Moderate to strong associations were also observed between cognitive ability and some musical and linguistic measures (see Table 4). Therefore, to ensure that any links between musical and linguistic skills cannot be attributed to underlying cognitive factors, scores in both cognitive tests were controlled for in subsequent regression analyses.

**Table 4:** Correlations between musical tasks and tests of non-verbal ability (WPPSI-Block Design) and verbal memory (Digit Span).

Tests	WPPSI-Block Design	Digit Span
**Musical tasks**		
Pitch Perception	0.14	0.41^**^
Tempo Perception	0.27	0.16
Melody Perception	0.18	0.22
Rhythm Perception	0.36^*^	0.35^*^
Song Production	0.23	0.05
Synchronization	-0.14	-0.04
**Language tasks**		
Language Grammar	0.54^***^	0.58^***^
Phonological Awareness	0.25	0.56^***^


*^∗^*p* < 0.05, ^∗∗^*p* < 0.01, ^∗∗∗^*p* < 0.001.*

#### Predicting Linguistic Abilities Based on Musical Skills

##### Musical abilities and phonological awareness

A linear model was built, in which all measures of musical abilities, as well as age and gender, were entered as predictors and phonological awareness was treated as the dependent variable. As can be seen in Table 5 (Model 2a) Rhythm Perception and Synchronization were significant predictors of phonological awareness. An increase in the performance of rhythm perception and lower error in synchronization were associated with better phonological awareness. Given that Synchronization had a lower *beta* and *t-*value we tested whether a model where Synchronization is included (Model 2a) would show a better fit than a more parsimonious model where only Rhythm Perception is included (Model 1a). Results indicated that the models differed significantly (see Table 5), suggesting that the Synchronization variable significantly contributes to the model.

**Table 5:** Summary and comparisons between Models 1a, 2a, and 3a predicting phonological awareness.

	*β*	*t*	*p*	*R*^2^	*AIC*	*F*	*p*	Model comparisons
Model 1a				0.40	-24.57	20.56	<0.001	
Rhythm Perception	0.63	4.53	<0.001					
								Model 2a vs. Model 1a
Model 2a				0.53	-31.03	16.29	<0.001	*F*(1,29) = 8.72, *p* < 0.01
Rhythm Perception	0.57	4.40	<0.001					
Synchronization	-0.38	-2.95	<0.01					
								Model 2a vs. Model 3a
Model 3a				0.65	-38.58	17.38	<0.001	*F*(1,29) = 9.74, *p* < 0.01
Rhythm Perception	0.44	3.77	<0.001					
Synchronization	-0.38	-3.41	<0.01					
Verbal Memory	0.36	3.12	<0.01					


The strong association between rhythmic and synchronization abilities and phonological awareness was further examined to determine whether this could be driven by latent cognitive factors accounting for better performance in both linguistic and musical tasks. Non-verbal ability and verbal memory were entered into the previous model (2a). The resulting model was highly significant (Model 3a in Table 5) and showed that Rhythm Perception, Synchronization and verbal memory significantly predicted phonological awareness, while non-verbal ability did not [*Beta* = -0.04*, t*(27) = -0.03, n.s.] and hence it was dropped. An increase in verbal memory predicted higher phonological awareness. Finally, the model that included verbal memory (Model 3a) was compared to Model 2a to ensure that verbal memory adds explanatory value to phonological awareness. Results showed that the two models differed significantly, thus proving the superiority of Model 3a over Model 2a in explaining the variance in phonological awareness.

##### Musical abilities and language grammar

In the final model (Model 1b), only Melody Perception significantly predicted language grammar (see Table 6), suggesting that better perceptual processing of melodies is associated with higher grammar scores. As shown in Table 6, Model 1b explained a notable amount of variance in language grammar scores.

**Table 6:** Summary and comparisons between Models 1b, 2b and 3b predicting language grammar.

	*β*	*t*	*p*	*R*^2^	*AIC*	*F*	*p*	Model comparisons
Model 1b				0.19	205.64	7.71	<0.01	
Melody	0.43	2.77	<0.01					
								
Model 2b				0.56	188.42	13.04	<0.001	
Melody	0.28	2.32	<0.05					
NVA	0.38	2.97	<0.01					
VM	0.35	2.71	<0.05					
								Model 2b vs. Model 3b
Model 3b				0.48	196.77	15.26	<0.001	*F*(1,32) = 5.38, *p* < 0.05
NVA	0.39	2.92	<0.01					
VM	0.44	3.26	<0.01					


*Melody, Melody Perception; NVA, Non-Verbal Ability; VM, Verbal Memory.*

Next, non-verbal ability and verbal memory were entered into the regression model (Model 2b). Results indicated that all variables significantly predicted language grammar (see Table 6). Finally, to ensure that Melody Perception adds explanatory value to language grammar, Model 2b was compared to a further model (Model 3b) where only non-verbal ability and verbal memory were included. Results showed that the two models differed significantly, suggesting that Melody Perception predicts language grammar, over and above general cognitive abilities. As shown in Table 6, Model 2b explained a significant amount of variance in language grammar scores and showed a better model fit compared to Model 3b.

Overall, results showed that there are strong associations between musical and linguistic skills in 3- and 4-year-old children. Crucially, we found unique associations between distinct musical and linguistic skills that remained significant even when cognitive skills, such as verbal memory and non-verbal ability were accounted for. Specifically, rhythmic measures (perceptual and sensorimotor) were the best predictors of phonological awareness while melody perception was the best predictor of grammar. This suggests that the mechanisms underlying these distinct music-language associations might be, at least in part, dissociable.

## Study 2: Effect of Informal Musical Experience at Home on Musical and Linguistic Skills

Study 2 was designed to examine whether informal musical interactions and experience at home (within the family) influence the children’s musical and linguistic skills as assessed in Study 1.

### Materials and Methods

#### Participants

Thirty-four parents of children who participated in Study 1 completed two self-report questionnaires on informal musical experience in the family and personal experience with music. Mean age of the parents was 36.4 years and in 44% of the families at least one parent had received a Bachelor’s degree or above.

#### Materials

The Musical Experience in the Family Questionnaire (henceforth MEF; provided by co-author of this manuscript FF) was used to assess frequency and type of musical engagement in the child’s home environment. The MEF included questions about frequency of musical engagement in the child’s home environment (singing and music making) as well as richness of musical exposure. The Goldsmith’s Musical Sophistication Index (henceforth Gold-MSI; Müllensiefen et al., 2014) was used to assess the parents’ degree of musicality. The average of maternal and paternal scores on Gold-MSI was entered in all analyses, together with the MEF score.

#### Procedure

At the launch of Study 1, parents of participating children were informed of the objectives of the project and the research procedure and a questionnaire including the materials described above was given to them. Completed questionnaires were collected at different times during the course of Study 1.

### Results

#### Associations of Children’s Musical and Linguistic Abilities With Musical Experience in the Family, Musical Sophistication of Parents and Parental Education

Bivariate correlations were performed between children’s musical perception and production tasks, composite scores of children’s language grammar and phonological awareness and the parental scores on Gold-MSI, MEF, and educational level. As can be seen in Table 7, a significant association was observed between the MEF scores and Song Production. Associations were also found between Gold-MSI and MEF scores indicating that parents’ engagement with music, combined with the amount of musical training they had received, can be reflected in the way they interact musically with their children. No significant associations were found between parental education and any of the music perception or production tasks, the MEF or the Gold-MSI.

**Table 7:** Correlations between MEF, Gold-MSI-Musical Sophistication, parental education, and musical tasks.

	MEF	Gold-MSI	Parental education
MEF	–	0.44^**^	-0.05
Gold-MSI	0.44^**^	–	0.07
Parental Education	-0.05	0.07	–
Pitch Perception	0.13	-0.11	-0.17
Tempo Perception	0.17	-0.03	-0.06
Melody Perception	0.02	-0.19	-0.02
Rhythm Perception	-0.03	0.16	0.18
Song Production	0.41^*^	0.33^1^	0.01
Synchronization	-0.17	0.23	-0.14
Grammar	0.36^*^	0.37^*^	0.14
Phonological Awareness	0.18	0.30	0.26


*^∗^*p* < 0.05, ^∗∗^*p* < 0.01. ^*1*^Marginally significant (*p* < 0.1). ^*2*^Composite scores of grammar and phonological awareness have been included. ^*3*^Since phonological awareness scores were not age-normed, age in months was controlled for in all relevant correlations. ^*4*^Preliminary non-parametric comparisons indicated that there were no significant differences between children receiving musical or dancing training outside the nursery/home (*n* = 5) and the rest of the sample (*n* = 29) in the linguistic composite scores, the MEF or the Gold-MSI. No significant differences were found for any of the musical tasks apart from Rhythm Perception where non-musically trained children performed higher than their counterparts (*U* = 26.5, *p* = 0.031).*

Interestingly, apart from the link between the MEF and song, and a non-significant trend for an association between the Gold-MSI and song (*p* = 0.062), no relationships were found between home musical environment variables and the development of musical abilities.

Both the MEF and the Gold-MSI were associated with language grammar scores (see Table 7), indicating that an enriched home musical environment may contribute to supporting the development of young children’s complex language skills. Interestingly, no associations were found between level of parental education and scores on the linguistic abilities tasks. Therefore, parental education was not included in subsequent regression analyses.

#### The Role of Informal Musical Experience in the Family in the Relationship Between Musical and Linguistic Abilities

Two linear regression models were built with language grammar and phonological awareness as dependent variables. Interactions between the musical abilities found to be the strongest predictors of these linguistic abilities and MEF were entered as predictors in each model separately.

##### Informal musical experience in the family, musical abilities, and phonological awareness

A linear model was built using phonological awareness as the dependent variable, and two interactions as predictors (MEF × Rhythm Perception, MEF × Synchronization).

Results indicated that both interactions significantly predicted phonological awareness (see Table 8). To ensure that the model where both interactions are included (Model 2c) shows a better fit than a more parsimonious model where only the strongest predictor is included (Model 1c), an ANOVA comparison was performed. Results indicated that the models differed significantly, suggesting that both interactions add notable explanatory value to the model (see Table 8).

**Table 8:** Summary and comparisons between Models 1c, 2c, and 3c predicting phonological awareness.

	*Beta*	*t*	*p*	*R^2^*	*AIC*	*F*	*p*	Model comparisons
Model 1c				0.22	-14.15	7.38	<0.01	
Rhythm:MEF	0.006	2.71	<0.05					
								Model 1c vs. Model 2c
Model 2c				0.45	-21.37	9.87	<0.001	*F*(1,24) = 9.76, *p* < 0.01
Rhythm:MEF	0.007	3.64	<0.01					
Synch:MEF	-0.012	-3.12	<0.01					
								Model 2c vs. Model 3c
Model 3c				0.59	-27.84	11.46	<0.001	*F*(1,23) = 8.48, *p* < 0.01
Rhythm:MEF	0.005	2.79	<0.05					
Synch:MEF	-0.017	-4.48	<0.001					
Gold-MSI		2.91	<0.01					


*Rhythm:MEF, interaction between Rhythm Perception and MEF; Synch:MEF, interaction between Synchronization and MEF.*

To examine whether these interactions would still predict phonological awareness above and beyond other important environmental factors linked to musical experience at home, the parents’ musical sophistication scores were entered into Model 2c. Results indicated that both interactions as well as the Gold-MSI significantly predicted phonological awareness (see Table 8). The model that included the Gold-MSI (Model 3c) was then compared to Model 2c. Results showed that the two models differed significantly, suggesting that all variables significantly and independently predict phonological awareness (see Table 8).

**FIGURE 3:**
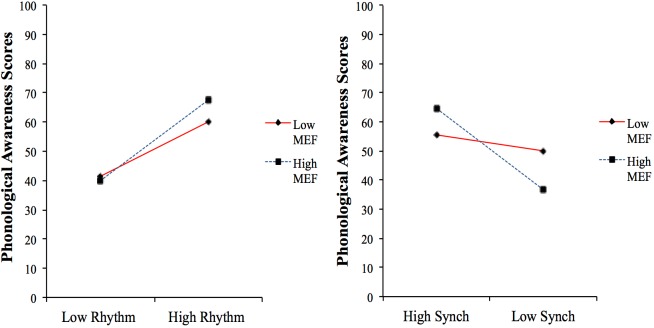
Interactions between MEF and musical abilities in predicting phonological awareness scores; Rhythm, Rhythm Perception; Synch, Synchronization (N.B. smaller scores in the Synchronization task indicate better performance). Means and standard deviations of all variables and unstandardized regression coefficients of both direct and interaction effects were used to generate points in the graphs that represent high and low performance in each of the independent variables.

To plot the effects of the interactions, two separate models were built with phonological awareness as the dependent variable and Rhythm × MEF interaction (Model 1d) and Synchronization × MEF interaction (Model 2d), respectively, as predictors. Both models significantly predicted phonological awareness [Model 1d: *F*(1,25) = 7.38, *p* < 0.05, *R^2^* = 0.22, Model 2d: *F*(1,27) = 4.32, *p* < 0.05, *R*^2^ = 0.13]. As can be seen in Figure 3, higher levels of MEF contribute toward a stronger link between the child’s musical abilities and phonological awareness.

##### Informal musical experience in the family, musical abilities, and language grammar

A linear model was built using language grammar as the dependent variable and the MEF × Melody perception as a predictor.

**FIGURE 4:**
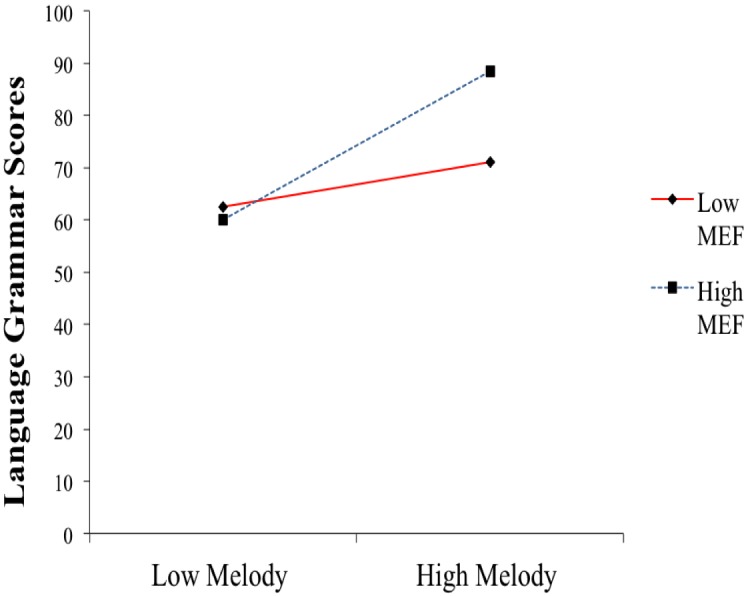
Interaction between Musical Experience in the Family and Melody Perception in predicting language grammar scores.

Results showed that the interaction significantly predicted language grammar (Model 1e) and explained a significant amount of the variance in language grammar (see Table 9). A plot of the effect of the MEF × Melody perception interaction on language grammar is presented in Figure 4. In line with what was observed for phonological awareness, the association between Melody perception and language grammar is stronger in children with higher levels of MEF.

**Table 9:** Summary and comparisons between Models 1e and 2e predicting grammar.

	*Beta*	*T*	*p*	*R^2^*	*AIC*	*F*	*P*	Model comparison
Model 1e				0.31	167.59	12.41	<0.001	
Melody:MEF	0.19	3.52	<0.001					Model 1e vs. Model 2e
								*F*(1,27) = 2.65, *p* = n.s.
Model 2e				0.37	166.76	7.91	<0.01	
Melody:MEF	0.17	3.12	<0.01					
Gold-MSI	0.33	1.63	n.s.					


*Melody:MEF, interaction between Melody Perception and MEF.*

The next step was to add Gold-MSI scores to the model. Results indicated that the interaction between MEF and Melody Perception still predicted language grammar significantly while the Gold-MSI did not (Model 2e, see Table 9). A comparison between Model 1e and Model 2e where all predictors were present showed that the two models did not differ significantly, confirming that the Gold-MSI did not explain a significant amount of variance in language grammar (see Table 9).

## General Discussion

The goal of the present study was to shed light on the relationship between language and musical skills in pre-school children. To this end, in Study 1 we tested the early links between distinct musical and linguistic skills. Three- and 4-year-old children underwent a thorough assessment of their musical and linguistic abilities. In Study 2, we examined associations between informal musical experience at home and early musical and linguistic skills as assessed in Study 1, aiming to test whether this type of experience mediates the relation between musical and linguistic skills.

### Strong Link Between Musical and Linguistic Skills in Early Development

We provide compelling evidence that both melodic and timing aspects of musical processing are associated with measures of phonological awareness and grammar in children as young as 3–4 years of age. Timing abilities and specifically tempo and rhythm perception and synchronization to a beat were consistently associated with phonological awareness. In contrast, melodic processing was particularly associated with language grammar.

Regression analyses revealed that synchronization to the beat (its variability) and rhythm perception were the most significant predictors of phonological awareness. These musical predictors contributed to phonological awareness above and beyond cognitive skills, namely, verbal memory, and non-verbal ability. These findings demonstrate that there are strong links between timing skills and phonological awareness as early as at 3 years of age, as previously reported for older children (5-year olds: Anvari et al., 2002; Verney, 2013; 8- to 13-year-olds: Huss et al., 2011). This is in line with the only study to date that showed an association between mutual synchronization in 3- and 4-year-old children and early reading skills (Woodruff Carr et al., 2014). The present study provides the first evidence that this link also extends to rhythmic pattern perception in children younger than 4 years.

A specific mechanism implying direct links between the perception of metrical structure in language and in music has been proposed by Goswami (2011; Temporal Sampling Framework) to explain specific phonological deficits in developmental dyslexia. According to the Temporal Sampling Framework, speech perception relies on the encoding of temporal modulations across different frequencies relevant for speech. Poor speech segmentation skills in dyslexic children are thought to arise from a specific difficulty in tracking the sound “rise time” (i.e., the time taken for the sound to reach its peak amplitude). Rise times are critical for segmenting the speech signal into syllables, as they reflect the patterns of amplitude modulation marking the passage from one sound to another. According to Goswami (2011), networks of neurons might entrain to an input rhythm marked by syllable rise times in speech, which form patterns of strong and weak beats alternating to avoid stress clashes (see also Giraud and Poeppel, 2012 for insights on the role of neuronal oscillations in speech processing). Temporal periodicities and meter are much more regular in music than in language. However, a similar neural mechanism might be at work with metrical structure in music as this also relates to strong and weak beat patterns (Goswami et al., 2013). This idea implies a specific and unique association between the processing of metrical structure in language and music and phonological awareness (Goswami, 2011). Our findings are consistent with the above framework, showing that timing skills such as the perception of rhythmic patterns within a melody and the ability to synchronize to an external beat are strong predictors of phonological awareness.

Interestingly, rhythm perception and synchronization ability predicted phonological awareness independently from each other, suggesting that these timing skills might be dissociable, at least in part. The idea that synchronization to the beat, and rhythm/beat perception can be separated and potentially under the control of partly different mechanisms has received some support from recent single-case studies of beat deafness and poor synchronization, (Sowiński and Dalla Bella, 2013; Dalla Bella and Sowiński, 2015; Bégel et al., 2017a), and in a patient with brain damage (Fries and Swihart, 1990). The intriguing possibility that perception and action may represent a double dissociation in the timing domain suggests that separate pathways might underlie perceptual and sensorimotor timing skills (Bégel et al., 2017a). These abilities might relate to phonological awareness via distinct neural mechanisms. For example, Goswami (2011) proposed that the tracking of the amplitude envelope in speech that is crucial for perceiving individual phonemes relies on phase-locking of neurons to slow oscillations (1.5–7 Hz) in the auditory cortex, while the same neural mechanism has been proposed for perceiving rhythmic structure in music (Large, 2008). On the other hand, it has been argued that both synchronization and phonological skills depend on the accurate representation of timing in the subcortical auditory system (Tierney and Kraus, 2014). In support of this idea, both individuals who exhibit variability in moving to a metronome (Tierney and Kraus, 2013) and children with developmental dyslexia (Hornickel and Kraus, 2013) show poor subcortical processing of timing information as reflected in delayed responses to sound and greater trial-by-trial timing variability in the auditory brainstem. Therefore, the possibility of separate timing skills impacting language through different (cortical versus subcortical) pathways is well-documented.

Crucially, we have provided new evidence of a relationship between melody perception and language grammar, an association not previously observed in pre-schoolers or other developmental groups. This relationship persisted even when verbal memory and non-verbal ability were controlled. These results strongly suggest that similar auditory perceptual mechanisms may be responsible for both melody perception and language grammar, at least at this stage in development. In addition, these mechanisms might be partly independent from those underlying the association between rhythmic abilities and phonological awareness.

With regards to the mechanism underlying the unique association between melody perception and grammar, one possibility is that melodic aspects of prosody are particularly important for the acquisition and development of grammar. Indeed, it has been suggested that pitch changes in continuous speech appear to mark boundaries between different syntactic units (e.g., clauses and phrases), thus assisting the extraction of grammatical information (Speer and Ito, 2009; Brooks and Kempe, 2012; Xie, 2012). Consistent with this view, Cohrdes et al. (2016) showed that melodic discrimination in 5- to 7-year-old children was associated with the processing of emotional prosody in linguistic phrases, suggesting that this type of basic auditory skill may work to strengthen more fine-grained aspects of language.

Another possibility is that statistical learning, a mechanism thought to underlie the internalization of melodic patterns of one’s musical culture (François and Schön, 2014) as well as the acquisition of grammar (Saffran, 2003; Saffran and Wilson, 2003; Gómez and Lakusta, 2004), may play a crucial role for both melody perception and grammar at this specific stage of development. With respect to the role of statistical learning, Saffran (2002) showed that both school-aged children and adults rely on distributional information in the sequencing of words (i.e., type A words always preceding type B words) to learn an artificial grammar, while Gómez and Lakusta (2004) found that even 12-month-old infants can categorize words based on distributional information. As suggested by Saffran and Wilson (2003), infants and children may use statistical regularities to acquire different levels of structure in language and this can occur in a cascaded manner. In their experiment, 12-month-old infants were exposed to multi-word sentences organized according to a finite-state grammar where transitional probabilities of syllables were lower between words compared to within words. Results showed that infants learned to distinguish between grammatical and ungrammatical sentences, suggesting that they were able to segment words from continuous speech and then use this knowledge to acquire grammatical rules in an artificial language (Saffran and Wilson, 2003). Given that the acquisition of grammar is a long and complex process that continues into the late pre-school years (Brown, 1973; Brooks and Kempe, 2012), it is possible that 3- and 4-year-old children continue to rely, among other mechanisms, on the distributional properties of input speech to internalize the structures of their native grammar. At the same time, between the third and fourth year of age, children are in the process of internalizing melodic and harmonic structures from their musical environment (Corrigall and Trainor, 2009, 2013), a process that has been argued to rely on statistical learning, that is, extracting regularities from musical input (Tillmann et al., 2000).

Given that developmental theories have supported the idea that the acquisition of language is a gradual process moving from the awareness of language phonemes in infancy (Jusczyk and Aslin, 1995) to more complex skills such as recognizing the meaning and functions of words in sentences (Cohrdes et al., 2016), it seems plausible that distinct musical skills that rely on common learning mechanisms will develop in parallel and in a similar fashion. Indeed, accounts in which musical skill acquisition develops in a gradual manner have also been proposed (Welch, 1985; Dowling, 1999). The findings of the present study bring together these accounts to suggest that specific mechanisms may operate in different manners across development to underlie both the acquisition of distinct language and musical skills but also the connections between them. This is compatible with emergentist models of language development which suggest that infants recruit a number of mechanisms to acquire language while the weight of reliance on different mechanisms changes over time (Hollich et al., 2000; Hirsh-Pasek et al., 2004; O’Grady, 2005). Specifically, we propose that sensitivity to metrical structure in 3- and 4-year-old children may contribute both to the acquisition of phonological awareness in language and to skills necessary for perceiving and producing rhythm structures in music. On the other hand, as demonstrated above, pre-schoolers may make use of sensitivity to statistical regularities to process melodies as well as grammatical structures at this specific point in development. Although the extraction of statistical regularities from speech may also play an important role in phonological awareness, evidence has shown that infants as young as 6–8 months already track statistical distributions of sounds in continuous speech to extract phonetic categories (6-month-olds; Maye et al., 2002) and sequences of syllables (8-month-olds; Saffran et al., 1996). Therefore, 3- and 4-year-olds may primarily rely on other auditory and cognitive skills to refine their knowledge of phonological structure. Similarly, the internalization of regularities and enculturation in rhythmic patterns may take place earlier than in melodic sequence (Hannon and Trehub, 2005b; see also Corrigall and Trainor, 2009, 2013). For instance, 6-month-old North-American infants can detect variations in both Western and Balkan music meters equally well (Hannon and Trehub, 2005a,b), whilst 12-month-old infants are already facilitated by the isochronous meter typical in Western music when detecting rhythmic changes in musical sequences (Hannon and Trehub, 2005b). Thus, infants at 12 months may already be displaying a form of musical enculturation for rhythmic aspects. Broadly in support of these findings, it has been suggested that rhythmic skills such as rhythm discrimination (Anvari et al., 2002) and production of rhythmic structures may develop earlier than melodic skills (see also Tafuri and Villa, 2002). This may explain the unique association between melody perception and grammar at 3- and 4-years.

The notion of specific intersections between distinct language and musical skills is also consistent with theoretical accounts according to which there are shared resources but also dissociable features in the cognitive processing of speech and music (Patel, 1998, 2003; Saffran, 2003; Peretz, 2006; see also Asaridou and McQueen, 2013 for a review of this issue). For example, in his highly influential theory, Patel has proposed that the online memory process of integrating new items in unfolding sequences in language (i.e., sentences) and in music (i.e., chord progressions) may rely on common cognitive and neural resources (Patel, 1998, 2003) while linguistic and musical long-term memory systems may be independent (Peretz et al., 1994; Brown et al., 2006; Slevc et al., 2009). Indeed, a number of behavioral and neuroscientific studies in adults and children have supported the idea of shared online processing (Koelsch et al., 2002; Jentschke et al., 2008; Fedorenko et al., 2009; Jentschke and Koelsch, 2009; Sammler et al., 2009; Schön et al., 2010; Slevc et al., 2013; Kunert et al., 2015). Studies of brain damage has offered findings sometimes consistent with the shared resources approach (e.g., agrammatic Broca’s aphasia associated with failure to process musical syntax: Patel et al., 2008) but has also found dissociations (e.g., impaired processing of harmonic relations with linguistic syntactic processing intact, Peretz et al., 1994, or impaired grammatical processing of language with preserved musical syntax, Slevc et al., 2016).

Overall, although there is evidence suggesting that some aspects of musical skills are domain-specific, the use of domain-general mechanisms for the processing of music and the acquisition of musical skills is generally supported (Saffran, 2003; Peretz, 2006). The present study informs influential accounts such as the above, first by identifying specific connections between distinct language and musical skills in a younger age than previously studied, and second by proposing domain-general learning mechanisms responsible for these links.

### Informal Musical Experience at Home Contributes to Language Development

The results of this study provide compelling evidence that informal musical experience is associated with children’s development of language grammar. This suggests that higher levels of home engagement with singing, music making and greater exposure to music can serve as scaffolding for the acquisition of verbal skills, greatly extending previous suggestions (Putkinen et al., 2013; Williams et al., 2015). Perhaps the rhythmic and melodic properties of music when combined with speech in everyday interactions offer additional cues for children to successfully extract and internalize linguistic structures and information from their environment. Indeed, infants as young as 6- to 8-months old appear to benefit from complex input (i.e., melody and lyrics) with information from one modality facilitating learning in the other (Thiessen and Saffran, 2009). Furthermore, given that musical interactions among groups in early childhood have been linked to pro-social attitudes and socio-emotional bonding (Custodero, 2006; Kirschner and Tomasello, 2010; Cirelli et al., 2014), music making in the home might facilitate emotion regulation and cooperation in the context of learning complex information. Indeed, it has been argued that affective and social aspects in the environment affect cognitive performance and learning in young children as well as infants (Kuhl, 2011; Franco et al., 2014). For instance, 9-month-old English-learning infants exposed to Mandarin native speakers who vividly interacted with them across 12 sessions learned to discriminate Mandarin phonemes, as opposed to a control group who was exposed to the same amount of foreign language sounds only via audio-visual and audio recordings (Kuhl et al., 2003). These findings emphasize the role of interpersonal interaction in language learning and cognition and suggest that the pro-social function of musical interactions may play an important role in promoting the acquisition of language. Whether it is predominantly socio-emotional functions of music-making that contribute to language learning or simply the perception-facilitating (e.g., rhythmic and melodic) properties of music, remains a crucial question for future studies.

When considering the influence of informal musical interactions at home on children’s musical skills, only the ability of children to sing in tune was significantly affected. This finding complements evidence on how immersion (i.e., contexts in which a child hears songs several times without being required to perform them), can facilitate song learning (Klinger et al., 1998). It may also be the case that children who often engage in singing activities at home might feel more comfortable about performing in a testing environment. Alternatively, the development of a range of musical skills might be influenced by additional variables that were not considered in this study, for instance personal engagement of the child with music, or quality of parent–child singing and music-making (e.g., singing in or out of tune, keeping a steady beat). It is also highly likely that unlike more focused music-making contexts (Gerry et al., 2012), the focus of these spontaneous interactions in the home is not to practice music in a consistent manner, but rather it is an opportunity for pleasant joint activities between parents and children (Custodero and Johnson-Green, 2003; Custodero, 2006). Not surprisingly, home musical interactions have been shown to play a supportive role to other learning goals (e.g., counting songs to learn the numbers), accompany everyday activities to make them enjoyable (Custodero and Johnson-Green, 2006; Barrett, 2009) or serve other purposes such as soothing, providing distraction and regulating behavior (Custodero and Johnson-Green, 2006; Young, 2008; Barrett, 2009).

Besides the direct effects of informal musical experience at home on either linguistic or musical skills in young pre-schoolers, the most intriguing and novel finding in the present study was that the interaction between home music experience and musical skills was predictive of language development. Specifically, the observed predictive relationships between children’s musical and linguistic skills, (namely the rhythm – phonological awareness and melody perception – grammar links), varied as a function of musical experience in the home, with children from more musically active families showing a stronger connection between musical and linguistic skills. This is consistent with findings by Forgeard et al. (2008) who found that predictive relationships between music perception skills and reading competence in 6-year-old children were stronger for musically trained compared to untrained peers. In our study, the fact that informal musical play often brings together speech and music within a context of positive interpersonal interaction may enhance interconnectivity between the areas of cognition that are engaged during this process. Such interconnections may later facilitate music to language transfer if children receive formal musical training. This finding complements insights provided by studies that have highlighted the role of active music making in promoting neural changes in the processing of language (Moreno et al., 2009; François et al., 2013; Kraus et al., 2014). Indeed, although actively engaging with music making appears to be important, it is also the unstructured and informal nature of home musical interactions that may have unique potential for cognitive transfer in the early years. Although this study was not designed to directly address these possibilities, the present findings generate critical questions for future exploration.

Finally, the level of parental education did not affect the relationships explored above, and the interactions between MEF scores and musical skills significantly predicted both grammar and phonological awareness skills even when the parents’ level of musical sophistication was taken into account. This means that it is specifically an active parent–child musical engagement that is mediating the music-language link. It is important to note, however, that the Gold-MSI had a contribution in predicting phonological awareness independent of the interaction between MEF score and musical ability, suggesting that the parents’ musical sophistication might also reflect a level of musical engagement with their children at home possibly not captured by the MEF Questionnaire.

## Conclusion

There are three conclusions from the research presented here. First, we have shown that distinct musical and linguistic skills are linked in young pre-schoolers, independently of individual differences in non-verbal ability and verbal memory. Thus, timing and melodic skills are not by-products of other cognitive skills, but rather they are independently associated with language development. *Rhythmic and melodic aspects of musical ability differentially predict, respectively, phonological awareness and language grammar*, revealing part of the developmental trend of the music-language relationships. This finding brings together theories of musical and linguistic development that support a step-by-step acquisition of skills in both modalities (Welch, 1985; Dowling, 1999; Cohrdes et al., 2016). Such distinct abilities rely on common learning mechanisms, yet partly dissociable domain-specific aspects. The task for future research is to identify the mechanisms underlying the connections between specific musical and linguistic skills along the developmental trajectory.

Second, we have provided evidence that *informal musical input at home can have an impact on the development of complex language skills* in 3- and 4-year-old children. This finding complements a growing body of research focusing on the potential of formal musical training for language development (e.g., Moreno et al., 2009; François et al., 2013; Kraus et al., 2014) and extends such promise to the informal home environment. It also suggests that informal and unstructured musical interactions may scaffold the development of other skills. More research is needed to delineate possible pathways through which engagement with music may support language, communication and social skills in the early years.

Third, above and beyond parents’ musical sophistication *musical experience in the family interacts with musical abilities in predicting language development*, thus suggesting that this type of experience may help to strengthen connections between musical and linguistic skills at a crucial stage when children develop school-readiness. This opens new areas of inquiry about the mechanisms through which musical experience may work to strengthen the developing brain. It also informs studies demonstrating the potential of musical training to promote changes in neurophysiological and cognitive functioning: it may be the case that a musically enriched home environment holds promise for enabling the transfer of skills from one domain to the other.

Together these results provide transferable insights by paving the way for using music-based training for strengthening language development in the early years. This is relevant for both early years practitioners and parenting practices, by suggesting musical enrichment as a powerful tool alongside other activities such as book reading, in supporting children’s development. Based on the present findings, musical programs focusing on specific skills could be designed to address language development targets at an age where the brain is still highly plastic. Such music-based interventions may have the potential to prevent language-learning difficulties, facilitate language development in challenging contexts (e.g., L2 acquisition in migrant children) and support language development in disadvantaged groups. For instance, training rhythmic skills may work to strengthen brain networks underlying the segmentation of syllables and words from the speech stream. Therefore, rhythm-based musical interventions could potentially improve phonological awareness and reading readiness in children at risk of developmental language disorders such as dyslexia (Overy, 2000, 2003; Goswami, 2011) or those being schooled in a new language. Such forms of rhythmic training can potentially take advantage of mobile technology and be developed into games or applications on portable devices, e.g., tablets or smartphones (Bégel et al., 2017b, 2018). In an age of rapid technological advance this can constitute a pleasant and highly motivating means for children to engage with interventions with promising potential for language development.

## Ethics Statement

This study was carried out in accordance with the recommendations of the Middlesex University Psychology Department’s Research Ethics Committee, with informed consent from all participants. All participants gave informed consent in accordance with the Declaration of Helsinki. The protocol was approved by the Middlesex University Psychology Department’s Research Ethics Committee.

## Author Contributions

NP conceived and designed the project, performed the experiments, analyzed the data, and drafted the manuscript. FF was involved in the design and planning of the research and supervised the project. SDB supervised statistical analyses. FF and SDB provided significant input that helped to shape the manuscript. NF performed the analysis of the synchronization data and contributed to the final version of the manuscript.

## Conflict of Interest Statement

The authors declare that the research was conducted in the absence of any commercial or financial relationships that could be construed as a potential conflict of interest.
